# Subject-specific finite element head models for skull fracture evaluation—a new tool in forensic pathology

**DOI:** 10.1007/s00414-024-03186-3

**Published:** 2024-02-22

**Authors:** Mikkel Jon Henningsen, Natalia Lindgren, Svein Kleiven, Xiaogai Li, Christina Jacobsen, Chiara Villa

**Affiliations:** 1 Section of Forensic Pathology, Department of Forensic Medicine, University of Copenhagen, Copenhagen, Denmark; 2 Division of Neuronic Engineering, KTH Royal Institute of Technology, Stockholm, Sweden

**Keywords:** Finite element analysis, Forensic pathology, Skull fracture, Computed tomography, 3D model

## Abstract

Post-mortem computed tomography (PMCT) enables the creation of subject-specific 3D head models suitable for quantitative analysis such as finite element analysis (FEA). FEA of proposed traumatic events is an objective and repeatable numerical method for assessing whether an event could cause a skull fracture such as seen at autopsy. FEA of blunt force skull fracture in adults with subject-specific 3D models in forensic pathology remains uninvestigated. This study aimed to assess the feasibility of FEA for skull fracture analysis in routine forensic pathology. Five cases with blunt force skull fracture and sufficient information on the kinematics of the traumatic event to enable numerical reconstruction were chosen. Subject-specific finite element (FE) head models were constructed by mesh morphing based on PMCT 3D models and A Detailed and Personalizable Head Model with Axons for Injury Prediction (ADAPT) FE model. Morphing was successful in maintaining subject-specific 3D geometry and quality of the FE mesh in all cases. In three cases, the simulated fracture patterns were comparable in location and pattern to the fractures seen at autopsy/PMCT. In one case, the simulated fracture was in the parietal bone whereas the fracture seen at autopsy/PMCT was in the occipital bone. In another case, the simulated fracture was a spider-web fracture in the frontal bone, whereas a much smaller fracture was seen at autopsy/PMCT; however, the fracture in the early time steps of the simulation was comparable to autopsy/PMCT. FEA might be feasible in forensic pathology in cases with a single blunt force impact and well-described event circumstances.

**Supplementary information:**

The online version contains supplementary material available at 10.1007/s00414-024-03186-3.

## Introduction

Autopsy and microscopy have remained the forensic pathologists’ most common tools for centuries [1]. With the introduction of post-mortem computed tomography (PMCT) to forensic pathology in the 1970s [2], a new tool became available. The forensic pathology community has since demonstrated how PMCT can be used for diagnostics, screening, identification, foreign object detection, 3D visualization, and 3D printing [3]. This paper aims to demonstrate how data from PMCT may be used for computer simulation of skull fractures to determine the plausibility of a proposed scenario.

The head is of great interest in forensic pathology as it often strikes objects or the ground in accidents and is an obvious target in assaults, and the brain is vulnerable to even small trauma [4]. Blunt force fracture of the neurocranium is seen in 5% of all forensic autopsies in Denmark [5], and almost all forensic cases of skull fracture have suffered lethal brain damage [6]. The skull fracture pattern may provide information on the shape and size of the object that caused the fracture, the number and order of impacts, and the force of the trauma, thus helping forensic pathologists infer the events leading to death [7]. Historically, forensic pathologists have relied on autopsy findings, scientific literature, experience, and available information on the proposed event when analysing skull fractures.

In 2006, finite element analysis (FEA) was introduced in forensic medicine [8, 9], giving forensic pathologists a new, potentially useful tool for skull fracture analysis. FEA is a computational technique often used in engineering to model and analyse complex systems by discretizing objects into a finite number of small elements and solving a series of partial differential equations [10, 11]. FEA enables objective evaluation of the probability of proposed traumatic events. Most head finite element models (FEMs) were developed for research focused on improving traffic safety or preventing traumatic brain injury, and not for forensic investigations [12]. In forensic pathology, FEA of gunshot trauma [13] and blunt force head injury in adults have been attempted [8, 9] using the Université Louis-Pasteur head model [14] and the Royal Institute of Technology’s Kungliga Tekniska Högskolan (KTH) head model [9]. Apart from the study on subject-specific infant skull fracture by Li et al. [15] and the study by Kleiven with analysis of fracture occurrence and -pattern [9], most of the finite element (FE) head models were coarse by modern standards, being comprised of fewer and larger elements than more recent FE head models such as the head model named “A Detailed and Personalizable Head Model with Axons for Injury Prediction” (ADAPT) [16]. More recent attempts with more detailed models were made by Jia et al. [17] and Gao et al. [18]; however, none of these models were subject-specific. Combining PMCT and FEA allows for subject-specific analysis of skull fractures. The 2021 ADAPT head model developed by KTH Royal Institute of Technology is anatomically detailed, can be personalized, and may be feasible for forensic use.

The objective of this study was to evaluate the feasibility of FEA for skull fracture in adults with subject-specific 3D FEMs obtained from PMCT and only the information available in routine cases as a tool in forensic pathology. For this, five cases of blunt force skull fracture involving falls from the department’s routine cases were simulated. Subject-specific versions of the ADAPT FEM, originally presented in [16], and later enhanced with an improved strain-rate dependent cranial material model [19], were used.

## Methods

This section describes how suitable cases were identified, the necessary information about the traumatic event was determined, subjects’ PMCT images were segmented, segmented subjects’ images were morphed to the ADAPT model leading to subject-specific FE head models, and FEA performed.

### Case identification

In a prior study, we had established a database of 250 deceased persons with blunt force skull trauma who were autopsied at the Department of Forensic Medicine at the University of Copenhagen between 2013 and 2019 [20]. From this database, we identified five cases suitable for FEA. The following criteria were used: (1) the deceased was an adult, (2) PMCT data of the head were available, (3) the event had only a single impact and a relatively simple skull fracture, (4) autopsy reports allowed assumptions of body movement at the traumatic event to estimate impact point and kinematics, and (5) police reports allowed event reconstruction to substantiate impact point and kinematics. Though the FEA itself is objective, the inclusion criteria 3 through 5 necessitated a partly subjective evaluation prior to analysis, the consequences of which will be discussed.

### Cases, traumatic events, and scene parameters

The cases are described superficially to avoid identification, and only information relevant to the traumatic event and scene parameters is described here. Four skulls had simple, linear fractures in the occipital bone caused by falls from standing or low-speed traffic accidents. One case had more extensive fractures of the frontal bone from fall from height. In three cases, the cause of death was acute ischaemic heart disease or subarachnoid haemorrhage due to vascular malformation. In three cases, the deceased fell limp to the ground from a standing position and these impacts resulted in the cranial fractures, whilst in the remaining two cases, death was a result of injuries sustained in the traumatic event.

From a combination of PMCT, police reports, autopsy reports, and hospital records, if such were included in the police reports, a junior doctor and an experienced forensic pathologist inferred the events at injury and impact point to the head. Subsequently, and in line with generally accepted methods, the impact angle and velocity of the head were determined with personalized FE human body models [21, 22], accounting for age, sex, height, and weight [19]. The subject information is summarized in Table 1.

**Table 1:** Characteristics of cases and traumatic events. *Only those relevant to the event are listed

Case	1	2	3	4	5
Manner of death	Natural	Accident	Natural	Natural	Accident/suicide
Age (years)	> 60	> 60	> 60	> 40	> 60
Sex	Male	Female	Male	Male	Female
Traumatic event	Fall	Traffic/fall	Fall	Fall	Fall from height (140 cm)
Fracture location (bone)	Occipital	Occipital	Occipital	Occipital	Frontal
Fracture type	Linear	Linear	Linear	Linear	Linear, complex
External lesions*	None	Contusions on breast and thighs corresponding to bike handlebars and wheel Abrasion on the left elbow	Laceration on the back of the head, just left of midline	Abrasion on the ulnar side of left lower arm Abrasion on the dorsal side of left thumb	Laceration on the forehead, in the midline, 3 cm above eyebrows, and bridge of the nose Contusions on knees and left shin Abrasions on right shin, left knee, dorsal side of right forearm
Other injuries*	Scalp haematoma, 5 cm posterior to external acoustic meatus	Extradural haematoma	Scalp haematoma, left of midline, between external occipital protuberance and lambdoid suture	Scalp haematoma, left of midline, just above the superior nuchal line	Fractures of the facial skeleton and right humerus
Proposed event	Collapsing from natural death, falling backwards	Struck by bicycle whilst standing still, falling backwards	Collapsing from natural death, falling backwards	Collapsing from natural death, falling backwards	Forward fall on stairs, right arm cushions impact, face and forehead impact floor with knees and shins impacting lower steps
Impact point	Occipital bone, left of midline, corresponding to scalp haematoma	Occipital bone, left of midline, just below the lambdoid suture	Occipital bone, left of midline, corresponding to scalp haematoma	Occipital bone, left of midline, corresponding to scalp haematoma	Frontal bone, corresponding to laceration
Impact velocity (m/s)	y-axis: 1.07 x-axis: 5.46	y-axis: 13.80 x-axis: 3.62	y-axis: 0.80 x-axis: 5.63	y-axis: 0.97 x-axis: 5.58	y-axis: 0.00 x-axis: 4.45
Notes	Impaired motor function	None	Contra-coup lesion of right temporal lobe	None	None
Forensic question	Two falls at separate events	Determine liability	Unknown manner of death	Medical malpractice	Unknown manner of death

### PMCT data

PMCT data were acquired with a Siemens Somatom Definition (Siemens Medical Solutions, Forchheim, Germany) using parameters listed in Table 2. The tube current varied due to automatic dose modulation.

**Table 2:** PMCT scan parameters

kVp	mAs	FoV (mm)	Slice thickness (mm)	Pitch	Slice increment (mm)	Reconstruction algorithm
120	180–450	500	0.75 or 1.00	0.80 or 0.75	0.6	Sharp h60f/h60s

### 3D segmentation of PMCT

The first step was to generate subject-specific 3D head models with the layers: (1) combined dense connective tissue scalp (skin) and soft tissue scalp (fat), (2) outer and inner table of cortical bone, (3) diploë, and (4) intracranial volume. This was done by segmentation of the PMCT images using 3D Slicer (v.4.11) [23]. In cases with extensive injury, craniotomy, or foreign bodies such as pressure gauges, the contra-lateral side was used as a basis for segmentation by mirroring the CT images as symmetry between the two halves of the skull was assumed. The facial bones and facial soft tissues were not segmented, and a mass corresponding to the mass of the missing face was later added to the models for FE using a semi-automated approach described below.

### Subject-specific head model creation

**Fig. 1:**
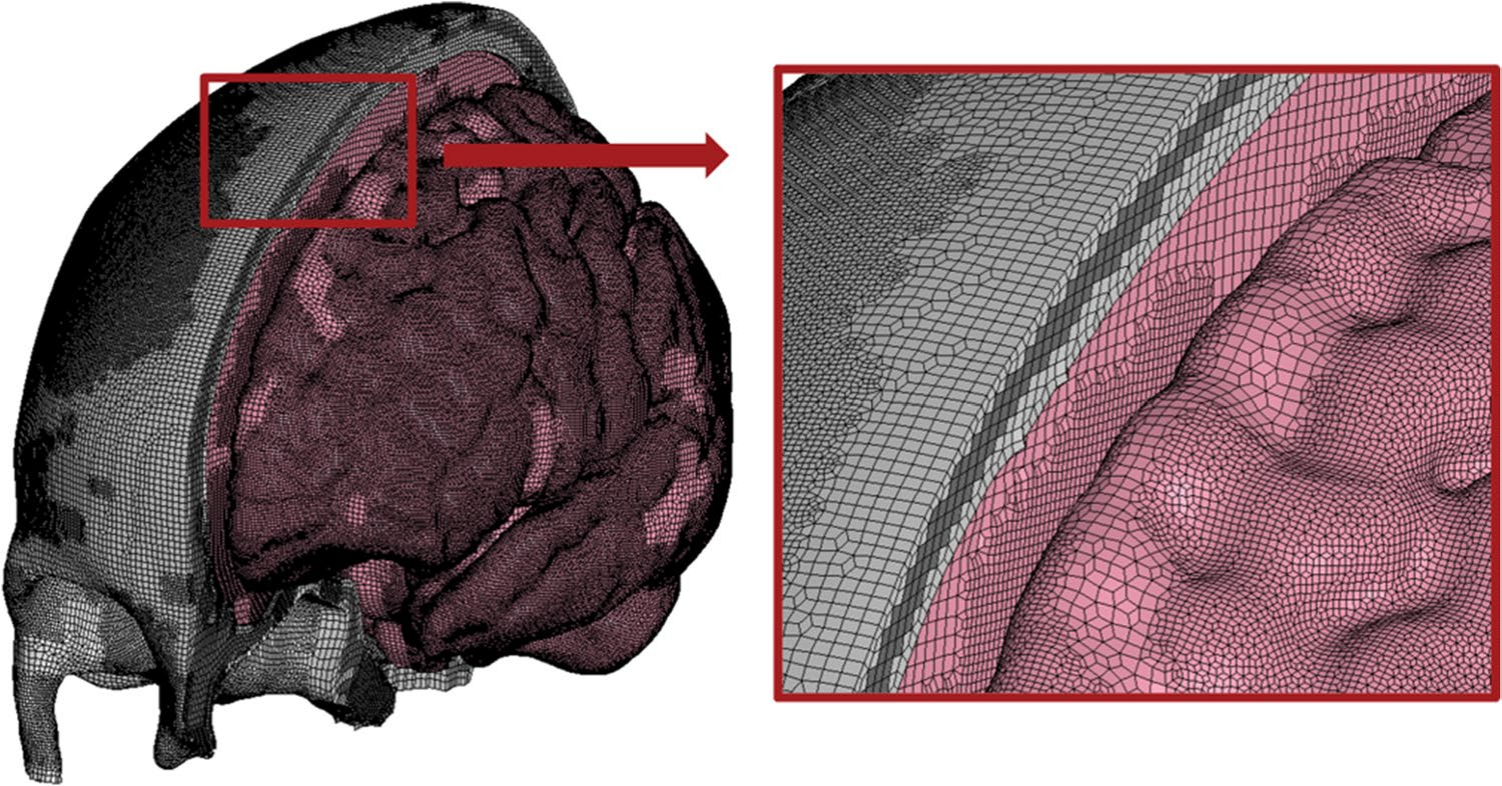
Baseline ADAPT FE head model with a sagittal cut through the skull (light grey) and diploë (dark grey) demonstrating cerebrospinal fluid and brain (pink). Cut-out demonstrates a closer view of elements

The above segmented subject-specific 3D head models were combined with the ADAPT FEM to create subject-specific FEMs that preserved the subject-specific geometry and thickness of the skulls. This combination of subject and FEM is referred to as *morphing* and the process has previously been described in detail by Li [24]. Summarizing the morphing process, the subject-specific models were aligned with the ADAPT model in 3D Slicer (v.4.10.2) and registered with the module BRAINSdemonWarp [25] from which displacement fields representing the anatomical difference between the subjects and the baseline ADAPT FE model were obtained. The displacement fields were then used to *morph* the baseline ADAPT, leading to subject-specific head models. The ADAPT FE model is based on the ICBM152, an “average” head constructed from MRI scans of 152 adults [26]. The baseline ADAPT FE head model is shown in Fig. 1, demonstrating the elements. The ADAPT FE head model includes cortical bone, diploë, meninges, cerebrospinal fluid, and distinct anatomy of the brain as a conforming, continuous mesh with hexahedral elements varying in size from 0.5 to 2.5 mm and a minimum Jacobian value of 0.45 [16].

### Finite element analysis and material properties

LS-DYNA (v.13: Livermore, CA, USA) and LS-PrePost (v.4.8: Livermore, CA, USA—Ansys LS-DYNA | Crash Simulation Software) were used for FEA and post processed in MATLAB (v2021a: The 251 MathWorks Inc, MA, USA). In the FEA, elements that reached their breaking point were deleted. For details, see Eqs. 1 and 2 in Lindgren et al. [27]. In all cases, the ground was modelled as an elastic material with parameters as specified in Table 3.

**Table 3:** Material properties of the ground

Elastic modulus	10 GPa
Poisson’s ratio	0.25
Density	2700 kg/m^3^

The ADAPT finite element model assumed the adult skull bone to be brittle, elastic-plastic, strain-rate dependent, and isotropic [19]. The scalp was modelled locally with two layers, the outer representing skin and the inner representing dense connective tissue with values previously used by Fahlstedt et al. [28]. We assumed neither sex nor age differences in the mechanical properties of tissues once individual thickness and geometry had been accounted for [29], and the sutures were not included in the ADAPT FEM. The material properties are described in detail in a previous publication [19].

## Results

### 3D segmentation and geometrical accuracy of the subject-specific models

**Fig. 2:**
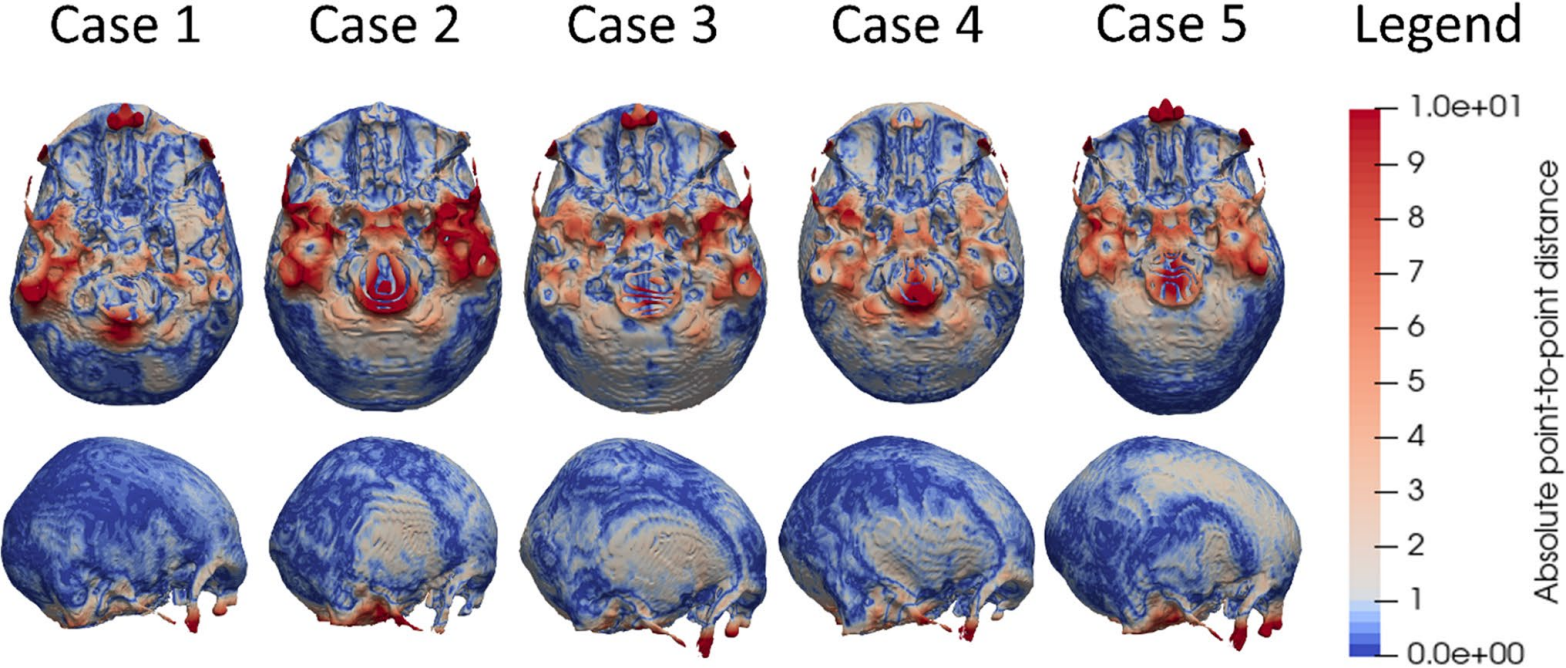
Point-to-point distance between segmentation and subject-specific FEM for case 1 to 5, lateral view and inferior view. Blue indicates a short distance and red indicates a longer distance

The relatively intact skulls were segmented with automatic tools in 3D Slicer in less than 5 h whilst the most damaged skulls required up to 15 h of additional manual segmentation. To quantify how well the subject-specific head models created through morphing represented the geometry of the real skulls (i.e. the segmented 3D PMCT), the point-to-point distance between the 3D PMCT model of each subject and the morphed subject-specific FE head model was calculated and visually demonstrated in a heat-map (see Fig. 2). Besides, DICE values were calculated to further quantify the accuracy, as presented in Table 4. Finally, the mesh quality of the subject-specific FE head models was quantified in terms of minimum Jacobian (Table 4), showing quality of subject-specific FE models comparable with baseline model quality.

**Table 4:** Mesh quality of the morphed subject-specific head models expressed as minimum Jacobian and DICE to quantify accuracy

	Minimum Jacobian	DICE excluding zygomatic bone and temporal styloid process	DICE
Case 1	0.19	0.88	0.81
Case 2	0.18	0.90	0.82
Case 3	0.31	0.89	0.82
Case 4	0.12	0.89	0.81
Case 5	0.14	0.87	0.84

### FEA of skull fractures for all cases

**Fig. 3:**
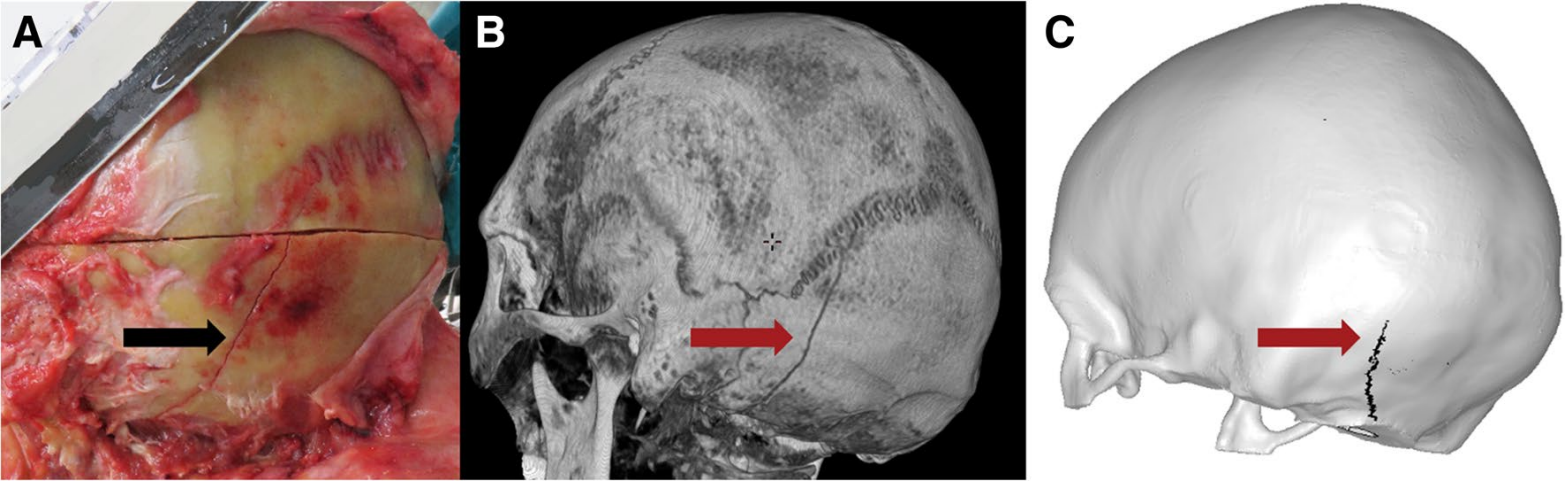
Case 1: posterior-sinister view of **A** autopsy photo, **B** PMCT 3D volume rendering technique, and **C** FEA with the predicted fracture marked as a black line. Fracture lines are marked with arrows. The real fracture line runs inferiorly from the lambdoid suture and terminates just posterior and left of the foramen magnum. The fracture predicted by FEA begins inferior to the lambdoid suture and runs inferiorly in a similar location to the real fracture and terminates just left of and posterior to the foramen magnum

**Fig. 4:**
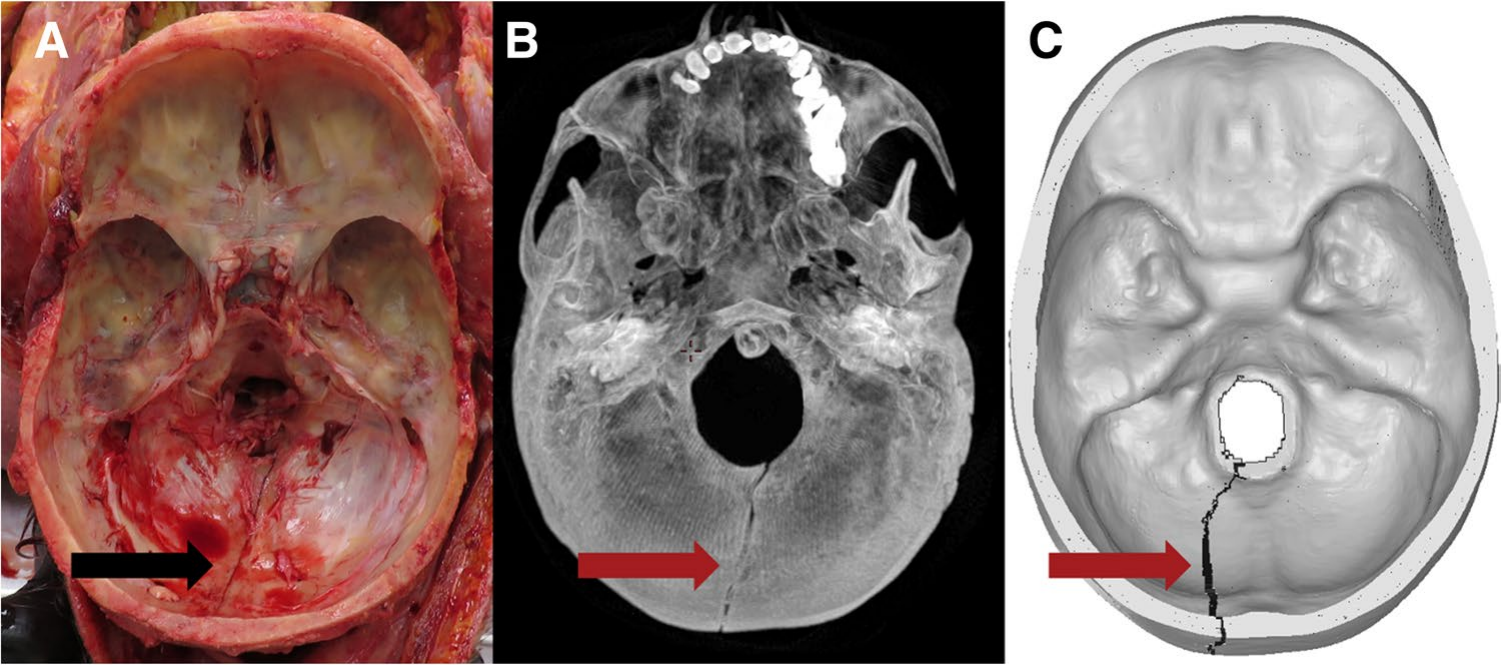
Case 2: superior view of **A** autopsy photo, **B** PMCT 3D volume rendering technique, and **C** FEA with the predicted fracture marked as a black line. Fracture lines are marked with arrows. The real fracture line runs inferiorly from just below the lambdoid suture into the skull base before just crossing the midline and terminating in the posterior margin of the foramen magnum. The fracture predicted by FEA originates in the same location as the real fracture and runs inferiorly to the foramen magnum, terminating in the posterior margin just left of the midline

**Fig. 5:**
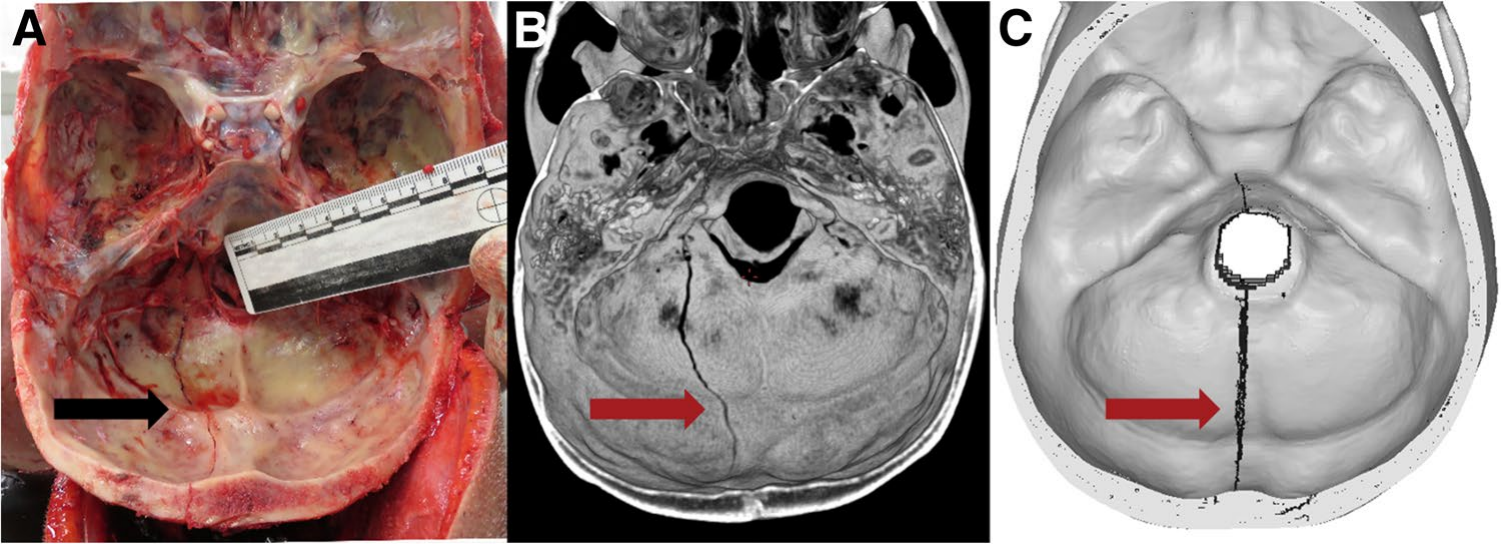
Case 3: superior view of **A** autopsy photo, **B** PMCT 3D volume rendering technique, and **C** FEA with the predicted fracture marked as a black line. Fracture lines are marked with arrows. The real fracture line runs inferiorly from below the lambdoid suture, left of the midline towards the skull base, tracing a path to the left from the internal occipital crest before continuing anteriorly to the left of the foramen magnum and into the middle fossae. The fracture line predicted by FEA is dissimilar in two ways: firstly, a short horizontal fracture line was predicted at the impact point; secondly, the predicted fracture extending towards the skull base runs in a straight line much closer to the midline and reaches the posterior margin of the foramen magnum before continuing from the anterior margin and into the middle fossae

**Fig. 6:**
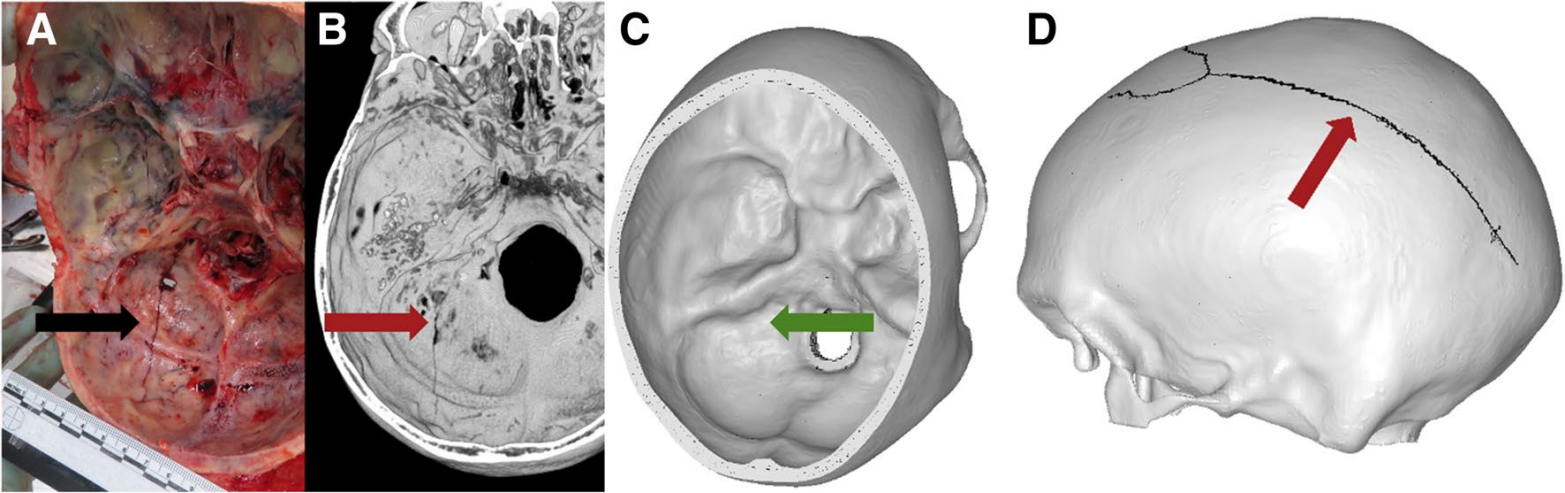
Case 4: superior view of **A** autopsy photo, **B** PMCT 3D volume rendering technique, **C** superior view of the skull base of FEA without predicted fracture, and **D** lateral view of FEA with the predicted fracture marked as a black line. Fracture lines are marked with arrows (red, black) and the “absent” fracture line marked with a green arrow. The real fracture line originates just above the superior nuchal line, left of the midline, and runs in a relatively straight line inferiorly into the skull base, terminating to the left of the foramen magnum. The predicted fracture runs anteriorly-superiorly, across the lambdoid suture into the parietal bone, and continues towards the left orbit in a semi-circle

**Fig. 7:**
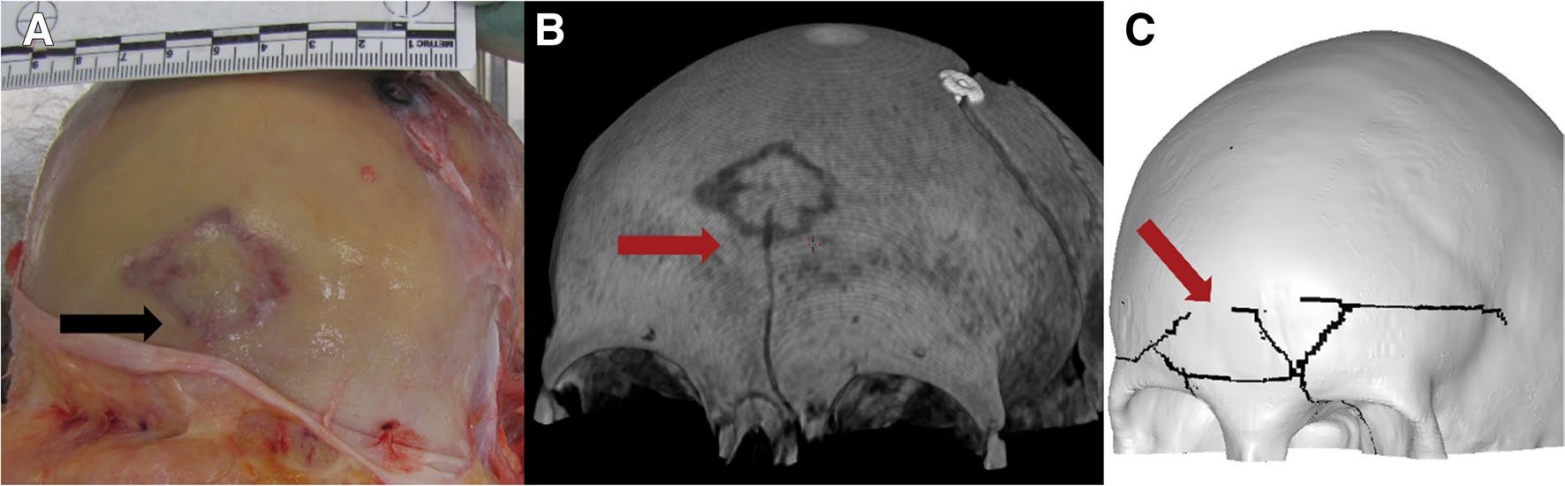
Case 5: anterior view of **A** autopsy photo, **B** PMCT 3D volume rendering technique, and **C** FEA with the predicted fracture marked as black lines. Note that partial healing and craniotomy are visible in A and B. Fracture lines are marked with arrows

**Fig. 8:**
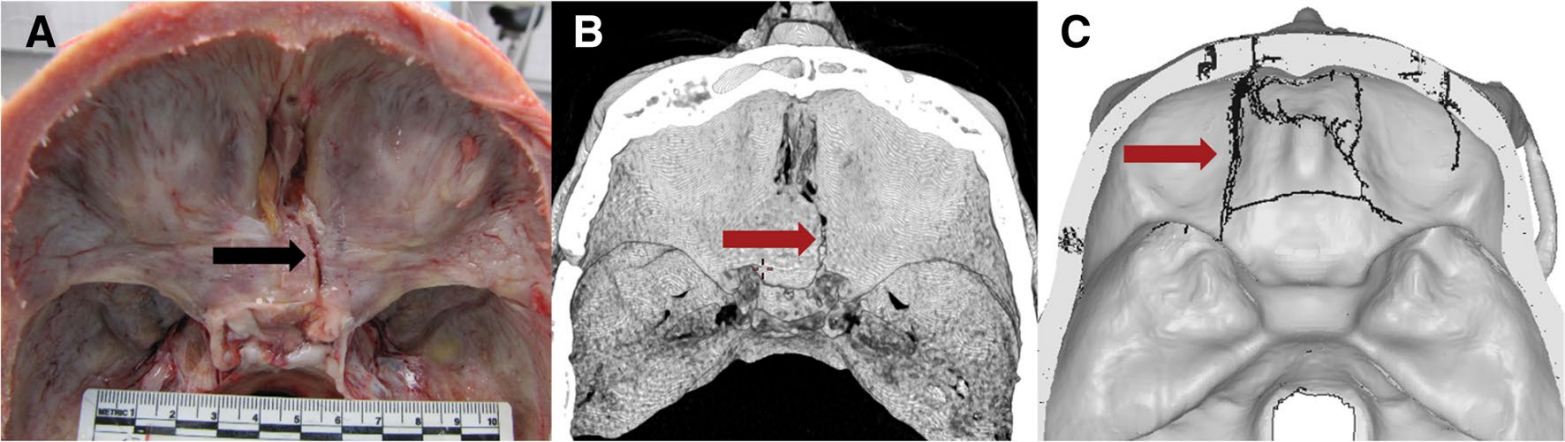
Case 5: superior view of **A** autopsy photo, **B** PMCT 3D volume rendering technique, and **C** FEA with the predicted fracture marked as a black line. Fracture lines are marked with arrows

**Fig. 9:**
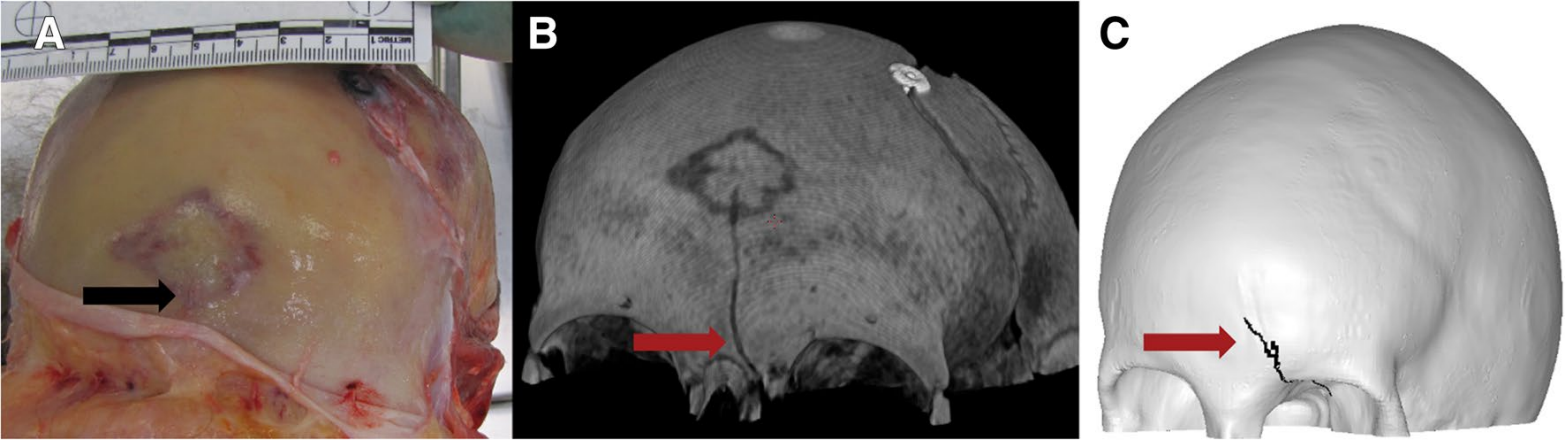
Case 5: anterior view of **A** autopsy photo, **B** PMCT 3D volume rendering technique, and **C** early time-step FEA with the predicted fracture marked as a black line. Fracture lines are marked with arrows. The real fracture lines are centrally in the frontal bone and shaped as a rhombus. A single, linear fracture runs from the middle towards the medial margin of the left orbit, continues in the base where it jumps the cribriform plate, and runs posteriorly on in the base of the right sphenoid. The predicted fracture resembles the lower half of a spider-web fracture in the vault part of the frontal bone with linear fracture lines extending from the impact point towards the right and left orbital margin and posteriorly in both parietal bones. The semi-circle of the predicted fracture has a larger diameter than the rhombus-shaped actual fracture

The finite element analysis of each case took about 6 h to complete on a supercluster with 128 cores. The results varied from correct fracture pattern in the correct location to the correct pattern in the wrong location to the wrong pattern in right location. Figures 3, 4, 5, 6, 7, 8, and 9 depict the fracture lines as seen at autopsy, on PMCT 3D volume rendering technique (VRT), and as simulated with FEA.

In cases 1 and 2, the fracture lines seen at autopsy and those simulated were nearly identical in location and pattern. In case 3, the simulation predicted a short horizontal fracture line at the impact point, and the simulation predicted a fracture line closer to the midline than the fracture observed at autopsy. The overall patterns of a linear fracture originating at the impact point, progressing into the skull base, and terminating in the middle fossae were comparable. In case 4, linear fractures of comparable extend and similar point of origin were predicted by simulation and seen at autopsy, however in different places, as the simulated fracture extended upwards in the calvaria and the fracture observed at autopsy ran in the skull base. In case 5, the simulation was initially identical to the fracture seen at autopsy, but the simulation progressed to a more severe spider-web pattern. FEA was performed in sequential time steps in the order of milliseconds, and if the FEA was stopped early, the initial simulated fracture resembled the actual linear fracture.

## Discussion

In this paper, we evaluated the feasibility of FEA of blunt force skull fracture in adults in forensic pathology. We used subject-specific 3D models generated from PMCT data and the information available in routine forensic pathology cases and demonstrated that it was possible to simulate fractures resembling the real fractures in three of five cases. We will discuss the methods, results, and the applicability of FEA in forensic pathology.

FEA in forensic pathology requires PMCT for two reasons: (1) to create the subject-specific FEMs, and (2) to help infer the circumstances of the traumatic event by drawing attention to potential bone fractures in the extremities and of the facial skeleton, which are locations not routinely dissected at autopsy [30]. To further substantiate the circumstances and head kinematics, information available in the standard police reports or documents included in the police reports such as hospital records and autopsy data are necessary, as external lesions such as abrasion, lacerations, and contusions and internal lesions such as fractures or dislocations may provide information that allows estimation of the kinematics and body movements during the traumatic event. For this study, we only included cases where the above-mentioned data were available; thus, the partly subjective inclusion criteria may bias the results favourably to FEA, as only cases assumed to be possible to simulate were included.

The segmentation process was automated as much as possible to obtain some objectivity, repeatability, and documentability. However, as with the subjective nature of input parameters, human decision-making in segmentation will influence the shape and thickness of the 3D head model. This decision-making is evident in dealing with e.g. partial volume effect, as also noted by de Kegel et al. [31].

The creation of subject-specific FE head models with hexahedral elements directly from PMCT data is challenging and time consuming [31]. Alternatively, an automated voxel-based solution may be used, but it results in jagged edges which in turn results in less accurate simulation. In this study, the subject-specific 3D models were created with a semi-automatic approach in 3D Slicer and then morphed with the ADAPT FEM, reducing the needed work time and maintaining relatively good accuracy as quantified with DICE (Table 4) and distance (Fig. 2) and thus potentially making subject-specific FEA more feasible in routine forensic pathology. A weakness of subject-specific head models based on post-injury CT is that intact 3D models without bone loss or foreign objects were necessary for FEA, as the inclusion of either would result in weaknesses causing a self-fulfilling fracture simulation. This makes FEA impossible in cases of severe fracture with displacement of comminuted fractures.

FEA requires a continuous shape to be discretized into a finite number of elements such as hexahedrals. The process of converting the irregular shape of the subjects’ skulls into a FE model into, e.g. hexahedral elements, is called *meshing*. The minimum Jacobian expresses the quality of the elements that constitute the FE model. Ideally, all elements should have the shape they were intended to, for example brick or hexahedral. When the elements are morphed to the shape of the subject-specific segmentation in the morphing step, some elements are geometrically distorted. The Jacobian ranges from − 1 to 1, where one represents perfectly shaped elements and values below zero indicate severely distorted elements. The DICE coefficient quantifies the difference between the segmented subject-specific images with image representing the ADAPT FE model, thus the resemblance between the subject’s skull and the subject-specific FE model. The DICE ranges from 0 to 1, where one represents a perfect fit between segmentation and subject-specific FEM, and zero represents a subject-specific FEM with no geometrical resemblance to the segmentation [32]. The minimum Jacobian in this study ranged from 0.12 to 0.31, indicating a relatively good quality of the mesh in the generated subject-specific models, helpful for stable running of FEA, and in line with commonly accepted values for “good” morphing and meshing [33]. The DICE varied from 0.87 to 0.90, indicating that the generated models accurately represented the subjects.

The bio-fidelity of the ADAPT FE head model used in this study was previously evaluated by Lindgren et al. against experimental test in uniaxial tension, skullcap indentation loading, and performed well in predicting fractures in terms of fracture initiation and propagation compared to real-world data [19]. This sets the skull model apart from other subject-specific head models [31, 34–36] of which only one has been evaluated against experimental data for fracture [35]. The assumptions regarding material properties of head models differ [31, 35], though most are valid and based on experimental testing of human bone. Naturally, the models cannot be expected to behave identically when based on different assumptions. However, this underscores that biological variation and a lack of consensus values may be a challenge to FEA. Subject-specific FE head models may be the first step in overcoming this challenge and subject-specific, heterogeneous bone density a further step.

In cases 1 and 2, and to some extent in case 3, the fractures were simple linear fractures, the event circumstances were well described, and the involved area of the skull had a low point-to-point distance between the subject and FEM. In these cases, fractures were correctly predicted by FEA. In case 4, the direction of fracture propagation in the simulated fracture was not consistent with the actual fracture. However, a linear fracture of comparable dimension was predicted. Skull fractures begin away from the impact due to bevelling and propagate back towards the impact point [37]. In the FEA, we used erosion meaning that elements were deleted as they reached breaking point. This deletion of elements influences the mechanical behaviour of the remaining elements, and the “wrong” fracture propagation may have been a self-fuelling process initiated by the deletion of the first wrongly predicted elements. Uncertainties regarding the initial angular velocities and the assumption of homogeneous skull properties for both cortical bone and diploë may partly explain the wrongful fracture propagation prediction. With only slightly lower bone density in the inferior parts of the occipital bone, the fracture might have propagated in that direction instead. A sensitivity study, substituting case 4 for cases with thinner skull bone for the occipital area, demonstrated fracture propagation inferiorly [19], which supports the speculation that case 4 might have had lower density of the occipital bone than assumed by the material model used. In case 5, the early time steps of the simulated fracture were largely consistent with the real fracture. However, the full simulation overpredicted the fracture and there was difference between the FEA and the real fracture. Based on the crack patterns observed by Gurdjian et al. in their stresscoat studies [38], we speculate that perhaps the force in the FEA was too high. As sensitivity studies showed, lesser force would have resulted in a less extensive fracture pattern [19]. In case 5, the deceased survived for an extended period, potentially masking some fractures as they potentially healed, thus artificially contributing to FEA “overpredicting”. The 3D segmentation assumed bilateral symmetry of the skull; thus, the potential effects of the craniotomy on subject-specific FEM generation could be ignored. The differences in fracture patterns between the simulation and the real fracture may be a result of the uncertainties regarding how much the deceased cushioned the fall and how much force the face and facial bones absorbed. In our simulation, no such absorption by the face was assumed. Cases 3, 4, and 5 underscore the partly subjective nature of the otherwise objective FEA in that segmentation and input parameters are influenced by human decisions. However, these are explicitly documented. But even with access to accurate kinematic measurements from controlled experimental studies, it has proved difficult to correctly predict fracture lines [31]. In this study, the initial head kinematics were derived from subject-specific multi-body models positioned in accordance with information gathered from police reports and autopsy [19]. As such, common for all studies is the approach of an educated guess for initial kinematics [8, 9, 17, 18]. Considering the uncertainty regarding the kinematics in forensic cases, great caution should be taken when using finite element analysis in a forensic setting, as even small changes in model parameters and event kinematics change the outcome of the simulation markedly [27, 31, 39–43], and even though FEA is on the cusp of potentially aiding forensic pathologists, technical challenges still warrant caution when interpreting the results. However, the assumptions and decisions are explicitly documented in the FEA and thus subject to scrutiny, discussion, and revision.

Jia et al. presented the results of the simulations as time-stress curves and values indicated that the stress from contact with the ground could cause fractures whilst the force from contact with the car was insufficient to cause any fractures. Jia et al. did not present the actual fracture seen at autopsy but noted that FEA demonstrated a linear fracture in the occipital bone in “about the same spot” [17]. Gao et al. concluded that FEA may be used for fracture line determination, but did not provide images for comparison of actual fracture and simulated fracture [18]. In Kleiven’s study, the fracture pattern was not investigated per se, and the results were ambiguous, demonstrating that a fracture could occur but would most likely not [9]. Raul et al. noted that “The anatomical distribution of the deleted elements is close to the fracture seen at autopsy, yet is not very accurate”. [8]. The ADAPT FE head model used in this study consists of about 4,950,000 elements, making it one of the FE head models with the most and smallest elements, allowing for very detailed fracture propagation prediction. Additional elements would result in a definite increase in computation time. It has been argued that simplified models could be sufficient [44], though it has also previously been argued that more elements may improve the quality of FEA [45]. Coarse FE head models with fewer and lager elements may be sufficient to predict if fracture occurs, but finer FE models with more and smaller elements are necessary for fracture propagation prediction. The downside to fine models is the need for significant computational capabilities such as the need for high-performance computing facilities. The question remains, given the inherent assumptions regarding event kinematics, whether FEA should be used for fracture pattern prediction or only for predicting fracture occurrence.

Potential applications of FEA in forensic pathology may include cases of multiple head traumas to determine which traumas could cause skull fracture. This was relevant in case 1 where the cause of death was natural, and the skull fracture occurred during the events leading to death. However, the deceased had also fallen prior to death and this first fall had not caused a skull fracture. Raul et al. simulated a comparable scenario, trying to distinguish between two falls as cause of death [8], and Jia et al. had previously investigated whether a motorcycle crash or subsequent collision with a car caused skull fracture [17]. In case 1, other evidence and autopsy findings allowed the determination of which fall had caused the skull fracture, but in cases in which other objective findings do not allow this determination, then FEA could guide further investigation: should the simulation result in either no fracture, extensive fractures, or a markedly different fracture pattern, then the forensic pathologist might have to consider other scenarios than those proposed. In case 2, the speed of the cyclist who struck the deceased was known from the police investigation. However, FEA may be used to approximate the speed of the involved parties in accidents by varying input parameters until the simulated fracture resembles the fracture seen at autopsy. This use is comparable to a study by Deng et al. who determined the angle and velocity necessary to cause skull fracture in falls [39]. Likewise, in case 3, the forensic pathologist was asked to determine whether death was an accidental fall or natural death resulting in a fall. FEA of the skull assuming a “limp” person falling from standing could help demonstrate that the skull fracture could have originated from a fall with an impact to the occipital region due to acute ischemic heart disease, and not from a fall with higher energy or a fall where the person had managed to cushion the blow to the head. It may be difficult for laypersons to understand that a fall from standing can cause skull fracture, despite it being well described [46]. FEA may help close speculations about interpersonal violence and bring closure to the relatives. Though not demonstrating the actual fracture precisely in case 3, FEA indicated that this specific person could have suffered a skull fracture when collapsing from natural death.

## Conclusion

In this study on the feasibility of subject-specific FEA in routine cases of blunt force skull fracture in adults, we demonstrated that FEA may be used in select cases to augment the basis for forensic pathologists’ analyses and conclusions. FEA is currently only feasible in cases with well-described events with simple kinematics and relatively intact skulls. As our cases also showed, caution should be taken when using FEA in a forensic setting.

This study was limited by the assumptions regarding the kinematics of the traumatic events and the fact that the ADAPT model has not been evaluated against cadaver studies with known kinematics. Future studies may perform validation against experimental tests or continue to simulate real cases, preferably with known kinematics, of different impact locations and events with different trauma mechanisms. This will help clarify the “error rate” for FEA in forensic pathology. Traumatic events captured by CCTV or other forms of video would provide a means to obtain more certain kinematics.

### Supplementary information

Below is the link to the electronic supplementary material.

ESM 1 (DOCX 22.0 KB)

## Data Availability

The data on cases are not available due to confidentiality.

## Code availability

The code for morphing is available through [24].
