# Supplementary material for: Subject-specific finite element head models for skull fracture evaluation—a new tool in forensic pathology
Source: Int J Legal Med. 2024 Feb 22;138(4):1447–58. doi: 10.1007/s00414-024-03186-3 (PMC11164801; doi:10.1007/s00414-024-03186-3)
Supplement: Supplementary file 1 — (DOCX 22.0 KB) [file 414_2024_3186_MOESM1_ESM.docx]

Appendix – Segmentation template

The PMCT images of all cases are saved in a dicom database in 3D Slicer.

The bone window / H60 kernel images are loaded.

With the “crop volume” tool, the smallest possible region of interest (ROI) still containing the cranium and soft tissue of the head is created and saved as “case_xx_cropped”.

In the module: ”Segmentation Editor”

The facial bones are not subjected to later finite element analysis, and the soft tissue rendering may enable identification. Therefore, part of the face is cut away.

Segment: **Face_cut_away**

Tool: Scissors

Settings used in “Masking” dialog:

Editable area: Everywhere

Modify other segments: Overwrite all

Fill inside, free hand.

Approach: On sagittal slices, identify the most anterior parts of the neuro-cranium. Draw a semi-circle outside the cropped area so that the straight line drawn by the tool between the start and end of the semi-circle is anteriorly to the neuro-cranium.

Segment: **Air**

Tool: Threshold: Lowest possible value : -145 HU (e.g. -2478 HU : -145 HU)

Settings used in “Masking” dialog:

Editable area: Outside all segments

Modify other segments: Overwrite all

This creates the “air segment” outside of the cranium, in the sinuses, in the mastoid, and in the brain and soft tissue where post-mortem gas development has occurred. We are not interested in keeping the air in the soft tissue and brain. To remove this:

Segment: **Air**

Tool: Islands

Remove small islands, minimum size 2000 voxels

Settings used in “Masking” dialog:

Editable area: everywhere

Modify other segments: Overwrite all

Segment: **Cortical_bone**

Tool: Threshold: 150 HU : highest possible value (e.g. 150 HU : 3661 HU)

Settings used in “Masking” dialog:

Editable area: Outside all segments

Modify other segments: Overwrite all

This range will capture most bone, except perhaps the roof of the orbit, parts of the mastoid and other thin structures. It will also create specked segments in the soft tissue and brain. These, we want to remove. We want to automatically “repair” as many gaps and holes in the cortical_bone segment as possible. These gaps may have occurred in the diploë, the roof of the orbit etc.

Segment: **Cortical_bone**

Tool: Islands

Keep selected island – click the skull

Settings used in “Masking” dialog:

Editable area: everywhere

Modify other segments: Overwrite all

Segment: **Cortical_bone**

Tool: Smoothing

“Closing (fill holes)”, kernel size 3.00 mm

Settings used in “Masking” dialog:

Editable area: Outside all segments

Modify other segments: Overwrite all

Segment: **Intracranial_volume**

Tool: “Level tracing”

Settings used in “Masking” dialog:

Editable area: Everywhere

Editable range: -250 HU : 200 HU

Modify other segments: Allow overlap

Approach: On axial slices, mark the largest area inside of the cortical bone that the tool will create. Allow overlap with the intracranial side of the cortical_bone segment, the editable range will ensure that the inner table remains un-overwritten. It is sufficient to do this on every other / third slice. Repeat on sagittal and coronal slices if necessary (it probably is).

This leaves gaps between the segment and the cortical bone and gaps between segments. To remedy this we use the fill gaps and grow segment tools.

Segment: **Intracranial_volume**

Tool: Margin

Settings used in “Masking” dialog:

Editable area: Outside all segments

Apply to only “intracranial_volume”.

Grow 1.mm

Approach: repeat until the segments expands past the cortical_bone, then undo last step.

Segment: **Intracranial_volume**

Tool: Smoothing, closing

Editable area: Outside all segments

Apply to only “intracranial_volume”.

Kernel size: 3.00 mm.

Segment: **Tissue**

Tool: Threshold: Lowest possible value : highest possible value

Settings used in “Masking” dialog:

Editable area: Outside all segments

Modify other segments: Overwrite all

Segment: **Cortical_bone**

Tool: Paint

Settings used in “Masking” dialog:

Editable area: Everywhere

Modify other segments: Overwrite all

Approach: Manually touch-up defects in the segment, especially areas of thin bone.

Segment: **Fracture**

Tool: Draw

Editable area: Everywhere

Modify other segments: Overwrite all

Approach: Hide all segments for better view of images. Then draw the fractures manually on all slices.

Segment: **Foreign_object**

Tool: Draw

Editable area: Everywhere

Modify other segments: Overwrite all

Approach: Hide all segments for better view of images. Then manually draw metal screws, implants etc. on all slices.

Segment: **Diploë**

We wish to create a segment, diploë, which should be the area between the inner table and outer table of the cortical bone, regardless of attenuation.

Step 1: Copy the segment “cortical_bone” to new segment “temp”

Step 2: Shrink “temp” until it does not expand past the highly attenuated parts of the inner and outer table

Step 3: Create segment “diploë” – use the threshold tool and keep the editable area inside of segment temp. This should also keep areas of fused sutures as cortical bone.

Step 4: Delete “temp”.
